# Increasing Rigor in Online Health Surveys Through the Reduction of Fraudulent Data

**DOI:** 10.2196/68092

**Published:** 2025-08-21

**Authors:** Wen Zhi Ng, Sundarimaa Erdembileg, Jean C J Liu, Joseph D Tucker, Rayner Kay Jin Tan

**Affiliations:** 1 Saw Swee Hock School of Public Health, National University of Singapore, National University Health System, 12 Science Drive 2, #10-01, Singapore, 117549, Singapore, 65 91878576; 2 Yale-NUS College, National University of Singapore, Singapore, Singapore; 3 Health and Social Sciences Cluster, Singapore Institute of Technology, Singapore, Singapore; 4 London School of Hygiene and Tropical Medicine, London, United Kingdom; 5 UNC School of Medicine, University of North Carolina at Chapel Hill, Chapel Hill, NC, United States

**Keywords:** online surveys, web-based surveys, web-based research, data quality, data integrity, data validation, fraudulent responses, fraud, survey fraud, methodological rigor, recruitment strategies

## Abstract

Online surveys have become a key tool of modern health research, offering a fast, cost-effective, and convenient means of data collection. It enables researchers to access diverse populations, such as those underrepresented in traditional studies, and facilitates the collection of stigmatized or sensitive behaviors through greater anonymity. However, the ease of participation also introduces significant challenges, particularly around data integrity and rigor. As fraudulent responses—whether from bots, repeat responders, or individuals misrepresenting themselves—become more sophisticated and pervasive, ensuring the rigor of online surveys has never been more crucial. This article provides a comprehensive synthesis of practical strategies that help to increase the rigor of online surveys through the detection and removal of fraudulent data. Drawing on recent literature and case studies, we outline several options that address the full research cycle from predata collection strategies to validation post data collection. We emphasize the integration of automated screening techniques (eg, CAPTCHAs and honeypot questions) and attention checks (eg, trap questions) for purposeful survey design. Robust recruitment procedures (eg, concealed eligibility criteria and 2-stage screening) and a proper incentive or compensation structure can also help to deter fraudulent participation. We examine the merits and limitations of different sampling methodologies, including river sampling, online panels, and crowdsourcing platforms, offering guidance on how to select samples based on specific research objectives. Post data collection, we discuss metadata-based techniques to detect fraudulent data (eg, duplicate email or IP addresses, response time analysis), alongside methods to better screen for low-quality responses (eg, inconsistent response patterns and improbable qualitative responses). The escalating sophistication of fraud tactics, particularly with the growth of artificial intelligence (AI), demands that researchers continuously adapt and stay vigilant. We propose the use of dynamic protocols, combining multiple strategies into a multipronged approach that can better filter for fraudulent data and evolve depending on the type of responses received across the data collection process. However, there is still significant room for strategies to develop, and it should be a key focus for upcoming research. As online surveys become increasingly integral to health research, investing in robust strategies to screen for fraudulent data and increasing the rigor of studies is key to upholding scientific integrity.

## Introduction

Surveys are a key tool in research, offering insight into participants’ minds and allowing researchers to better understand their motivations and preferences [1]. Traditionally, survey data was collected primarily through paper questionnaires, potentially distributed through the mail or face-to-face, and eventually grew to include telephone surveys [2,3]. However, as use of the internet has grown and response rates for traditional survey modes dropped, the primary choice for surveys has shifted to online surveys, alternatively known as web-based surveys, which focus on distributing a survey form or instrument hosted on the internet, even though actual distribution methods can range from more traditional ways like mail to newer methods like social media advertising [2,4].

The use of online surveys in internet-based health research has become more common, largely due to its ability to efficiently reach a large audience in a cost-effective manner and its low barriers to participation [5,6]. For example, online surveys eliminate the need for face-to-face interaction and remove transportation and logistical barriers, offering participants the chance to complete surveys at their own convenience and comfort, reducing the burden on them [7,8]. Furthermore, researchers can easily reach underrepresented populations, as well as create samples that ignore geographic boundaries [9,10]. Depending on the design, it also gives participants the ability to remain anonymous and the freedom to respond honestly without stigma or judgment [11]. Online surveys can elicit significantly more reports on socially undesirable behaviors in comparison to surveys administered in person, highlighting the benefit of online surveys when it comes to sensitive topics [12]. COVID-19 has also accelerated the use of digital methods for research purposes, prompting the transition of surveys from in-person to online formats [13,14].

However, the rise of online surveys comes with an increased risk of fraudulent behavior [8], which can be defined as “individuals, groups or computer processes (ie, bots) participating in online, internet or web-based data collection methods at a statistically significant level, such that data are or would be measurably distorted” [15]. Fraudulent behavior comes in many forms, which leads to further complications when screening for it. For example, incentives are a popular method to encourage participation, but they increase both authentic response rates and repeated responders [16]. Individuals may choose to misrepresent themselves to match eligibility criteria (alias scammers) or submit multiple surveys in order to receive more incentives (repeat responders) [17]. However, incentives are not the only motivational factor, as response distorters could spam responses in an effort to misinform results due to their own agenda or political motivation [18,19]. There has also been an increase in easily obtainable sophisticated software applications, otherwise known as bots, which are designed to automatically fill out surveys, allowing individuals to quickly flood surveys with responses [20]. Furthermore, there are also careless responders, where individuals do not give sufficient attention to questions or fail to read them comprehensively, leading to answers that may not accurately reflect reality [21].

Another consideration when crafting an online survey is the sampling method, which has the potential to significantly introduce errors and bias into data collection, reducing the representativeness and generalizability of results [22]. When carrying out any research, it is important to clearly define the population of interest and tailor the sampling method to effectively engage that target population [23]. Sampling methods can largely be split into two categories: (1) probability samples, in which elements from a population are randomly selected and elements have a nonzero known probability of being selected, and (2) nonprobability samples, which are nonrandom and the probability of each element being selected may be unknown [1]. Probability sampling is generally a more accurate method to establish the characteristics of an entire population. In comparison, nonprobability sampling is typically used when access to the full population is limited or not needed, such as when researchers only want to focus on a specific subgroup of characteristics. If an inappropriate sampling method is chosen, researchers run the risk of collecting data that is not an accurate representation of the target population [24].

Despite the difficulty of doing so, ensuring that data collected is accurate is critical, as the inclusion of even a small amount of fraudulent data can create or mask statistically significant differences, decreasing the rigor of the study [18,25]. The validity and integrity of results then come into question and may even artificially create relationships between uncorrelated factors [26]. For example, a study found that of the 1281 respondents, only 197 were real respondents, and the inclusion of fake respondents into the results significantly altered the findings—both creating and masking relationships between factors [27]. Without a sufficiently rigorous data screening process, bias and noise could be introduced to the dataset through the inclusion of fraudulent data, leading to incorrect conclusions of study results and potentially biasing future research or policy recommendations [8]. Furthermore, fraudulent behavior not only compromises the quality of the research done but also increases research costs through the disbursement of incentives to individuals who had already completed the study or were ineligible and the need for increased time spent on identifying fraudulent responses [7,11].

**Figure 1.**
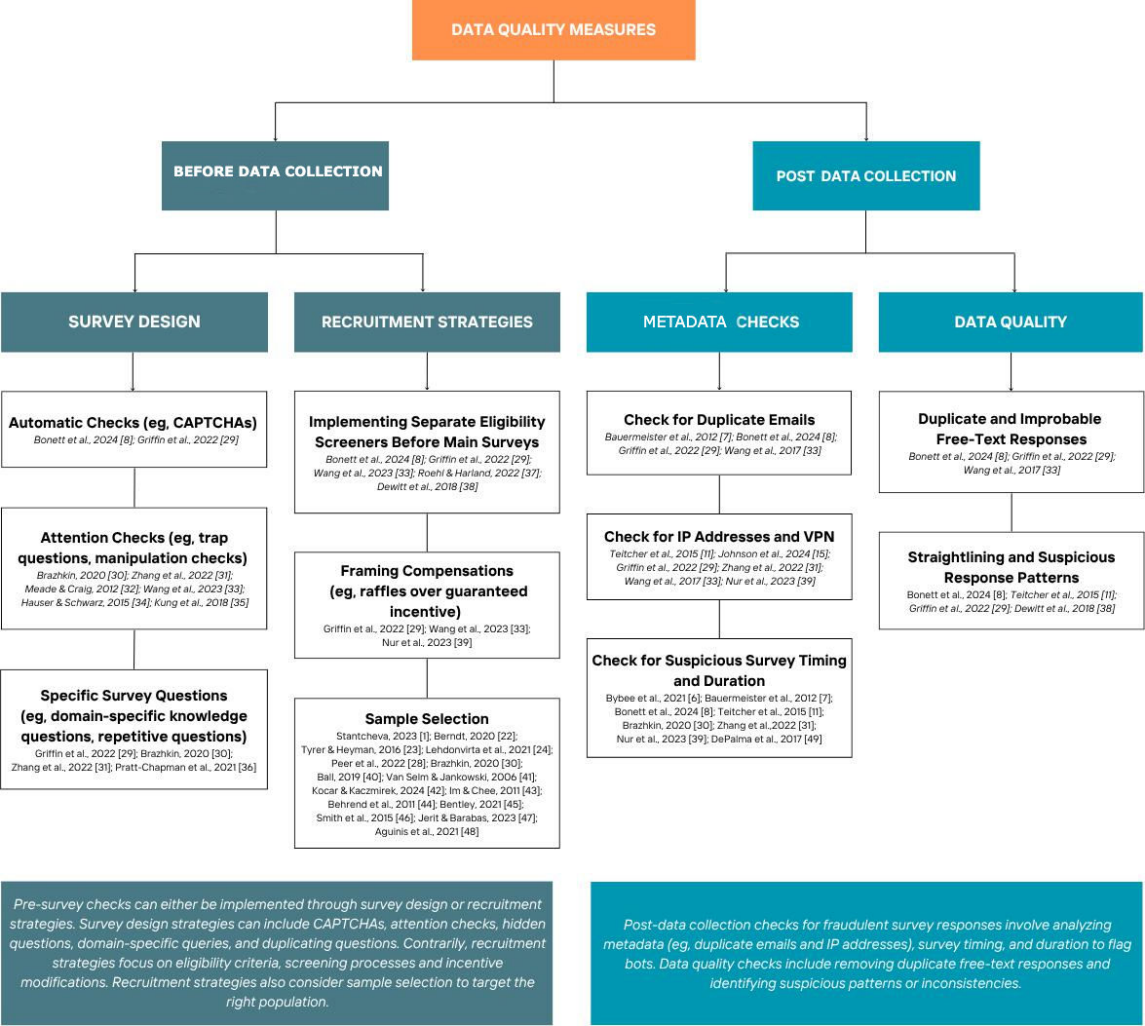
Measures to increase rigor of online health surveys [1,6-8,11,15,22-24,28-49].

Data quality comprises many different attributes, such as reliability, accuracy, and integrity; yet, different disciplines and platforms will give rise to different levels of importance placed on each attribute when conducting research [28]. One of the key threats to online surveys is the rise in fraudulent responses; yet, there is a lack of articles exploring the impact of fraudulent data on online surveys and how to best guard against them [27]. The purpose of this article is to examine the methods in which researchers can increase the rigor in online surveys by taking an integrated approach to recruitment, survey instrument design, and metadata checks in order to reduce the inclusion of fraudulent data. The measures that are commonly used by researchers now can be largely split into 2 different phases of research: before data collection and post data collection. Subsequently, each phase gave 2 main areas of focus, (1) survey design and (2) recruitment strategies for predata collection, and (3) metadata checks and (4) data quality for postdata collection. A summary of the methods mentioned in this article can be found in Figure 1.

## Predata Collection: Guarding Against Fraudulent Data

Presurvey methods that help to guard against fraudulent data can either be implemented through survey design or recruitment strategies. Survey design strategies are primarily meant to make screening for fraudulent data easier. Contrarily, recruitment strategies aim to prevent fraudulent responders’ access to the survey.

### Survey Design: Automatic Checks

One basic automatic check to protect against bots is CAPTCHAs, which is based on user actions such as checkbox clicks and image challenges [8]. Newer versions like Google’s reCAPTCHA V3 use sophisticated algorithms to evaluate user interactions based on various criteria, such as typing speed and IP address, which are then scored based on the study’s scoring system and expectations in order to identify possible bots [29]. Honeypot questions are an additional automated defense against bots that can be easily included within surveys alongside CAPTCHAs; these are questions that are designed specifically for bot detection and hidden from actual participants using custom code [8]. Thus, receiving answers to a honeypot question would indicate bot activity.

### Survey Design: Survey Questions

Another widely used strategy to identify and exclude inattentive respondents is attention checks. The most commonly suggested method is to ask for a specific response, otherwise known as a trap question or instructional manipulation check [30]. For example, participants could be asked to pick the italicized option [31], or a specific choice from a list, eg, please select the option “strongly agree” [32]. Current literature advises attention checks to be used throughout the survey to better identify responders who are giving low-quality responses [30]. The wording and quantity of trap questions should strike a balance between filtering out low-quality responses and ensuring that genuine respondents are not accidentally filtered out [32]. An alternative method is to make use of speed bump questions, which require participants to properly read through the question and use reason to arrive at an answer, filtering out bots or respondents who answer hastily or automatically [33]. Speed bump questions can look like this, “The man couldn’t lift his son because he was so weak. Who was weak, the man or his son?” However, researchers should consider the type of research they are doing before making use of attention checks, as they have been found to increase deliberation, potentially affecting cognitive task performance [34]. On the other hand, other research has found that they do not affect participants’ responses on scales or scale validity [35]. Researchers should thus monitor responses or trial survey questions to see if responses are significantly affected before rolling out their surveys officially.

Another strategy researchers can consider using is to leverage domain-specific knowledge to detect respondents who are not from the correct target audience. This is especially effective in fields where many industry-specific terms are used, and researchers can make use of this by including qualitative response formats [30,31]. For instance, one survey targeting experienced programmers included simple programming tasks and questions as assessments [30], while another survey used specific terminology and jargon as soft-checks to verify expertise [31]. Subsequently, identifying inconsistencies or suspicious responses compared to established norms, such as unlikely health symptoms for the given demographic, can identify unfit respondents [36].

Researchers have also found success in using duplicate questions presented in diverse formats. Researchers have advocated for requesting the same data point in multiple formats [29] or incorporating specific items into questionnaires that essentially ask the same question more than once [30]. For example, one study detected bot-based responses by including duplicate gender identity questions, which would trigger subsequent logic checks and reveal bots that followed code rather than survey logic [31]. Inconsistent responses to duplicate questions serve as indicators of low-quality responses, so researchers should take note of mismatches in answers. Researchers should also disguise these checks by distributing questions across different sections of the questionnaire and phrasing them differently to prevent easy identification.

### Recruitment Strategies: Eligibility Screening

To ensure the integrity of survey responses, researchers can implement rigorous eligibility screening processes and streamline recruitment procedures. For example, researchers can consider using 2-stage recruitment, in which participants first have a public eligibility screener to assess whether they meet eligibility criteria before a second personalized link is sent to them for the main survey [33]. The eligibility screener can further implement features like CAPTCHAs or browser cookies to prevent duplicate responses [33]. Eligibility screeners could also request contact information so that researchers can contact participants personally to confirm their identity or ask for documents that could confirm information critical to eligibility [37]. In order to prevent individuals from tailoring their responses to meet eligibility criteria, researchers can consider recruiting participants without explicitly disclosing eligibility criteria upfront [29]. Furthermore, having duplicated questions in the eligibility screener and main survey will allow researchers to look for inconsistencies in responses, which is another way to expose suspicious respondents [8]. Researchers can consider requiring participants to go through steps before accessing the survey, such as using specialized links or passwords, but must also ensure that this process is as straightforward and user-friendly as possible; otherwise, it risks increasing dropouts [38].

### Recruitment Strategies: Compensations

While incentives can promote higher numbers of survey responses, they also run an increased risk of attracting fraudulent responses [33]. To mitigate such risks, researchers can consider structuring participant reimbursements in a way that reduces the financial rewards of fraudulent responses without undermining overall study participation. For example, framing the incentive as a raffle instead of a guaranteed reimbursement reduced the number of bot responses from 633 to 23 in one particular study [29]. This may be explained by the way in which bots are programmed to seek out guaranteed financial incentive surveys, and thus avoid surveys that include random chance incentives [29]. Furthermore, researchers should appropriately match incentives to study requirements and payment norms in the area, as incentives that were seen as higher than others attracted more fraudulent responses [39]. Lastly, separating the reimbursement form and letting participants be directed to it only after finishing the main survey can help ensure only genuine participants receive the incentive [29].

### Recruitment Strategies: Sampling Methods

The way in which researchers derive their sample is key, as it is the basis for the entire study. Probability sampling is not typically used with online surveys, simply because it is difficult to establish a probability sample of the whole population on the internet; the population of interest may not necessarily be online, and even those online may not use the internet frequently enough to be captured [40,41]. However, there are workarounds if a probability sample is required, such as randomly sampling and contacting participation via another method (eg, telephone or mail) and doing the actual survey online, or defining a target population (eg, health care workers in a specific geographical area) and inviting all of them to complete the online survey [40]. While probability sampling often leads to a more representative sample of the whole population, it may not necessarily be the most ideal sampling method, as not all research requires the entire population, and it is difficult to obtain representative numbers of smaller subpopulations [23]. Instead, researchers can consider nonprobability sampling, which is ideal for sampling hard-to-reach or hidden populations, where the members are unknown and make up a small proportion of the whole population, meaning that it is difficult to get a sufficiently large sample through probability sampling [22]. Regardless of whether probability or nonprobability sampling is used, the majority of those selected to be sampled should complete the survey; if not, there is a high potential for the sample to be skewed in an unpredictable direction due to potential selection bias and the introduction of large amounts of sampling error [23].

One of the most common sampling methods used in online surveys is river sampling, which is a nonprobability method based upon convenience sampling. In river sampling, researchers place a survey link somewhere it is likely to be seen by members of the target population, such as a web page or through email [24]. However, while cost-effective and convenient, it suffers from coverage bias, where not every subgroup is represented equally on digital platforms, leading to results being most representative of active users of the chosen platform, and not necessarily the target population. It is a great method to quickly recruit large numbers of respondents, but is particularly vulnerable to attacks by fraudulent respondents due to the easy access to the survey [24]. Despite its limitations, river sampling can be very beneficial if the study is focused on a narrower population, such as undergraduate students at a university [1].

Another common sampling method used in online surveys is commercial panels, which can be either probability-based (eg, KnowledgePanel or AmeriSpeak) or nonprobability-based (eg, Qualtrics Panels and Dynata) [28]. Commercial online panel providers include participants who are prerecruited and have agreed to take part in research and differ based on how their participants are recruited. Probability-based panels tend to rely on “offline” recruitment, such as random digit dialing or address-based sampling to invite participants to join, while non-probability-based panels run on a volunteer or opt-in basis [42]. In general, commercial online panels are an alternative option that offer respondent pools that better mirror the demographic compositions of the general population compared to river sampling, but at a greater monetary cost [24]. Commercial panel providers ensure quality by verifying their respondents beforehand, placing the onus of respondent verification on them rather than the researcher [1]. Furthermore, researchers can impose quotas on specific demographic criteria, forcing participants to better approximate the target population. Researchers must keep in mind that even with quotas, online panel providers may still be unable to fully reach hidden populations, as subpopulations within the participant pool may differ in the frequency of how often they actually access the panel and complete surveys, resulting in results that reflect those who are most online rather than being fully representative of the target population [24,43]. The quality of online commercial panels also depends on how it is managed, and researchers should take care in checking how panel participants are recruited or managed before committing to a specific panel in order to maintain a high level of accuracy in the data collected [30].

Lastly, crowdsourcing platforms such as Amazon’s Mechanical Turk and Prolific serve as non–probability-based online marketplaces where potential participants are “hired” to participate in research, but differ from panel providers in that their participant pools are not necessarily curated [24,44]. This means that researchers need to expend extra effort in screening participants for specific target populations, and larger sample sizes are required in order to sufficiently power analysis [45]. Another benefit is that large sample sizes can be generated quickly compared to other methods. While not always ideal for research that requires a sample that is representative of the general population, researchers should leverage the unique strengths of crowdsourcing platforms when studying nondemographic subpopulations or hard-to-reach subpopulations [46]. Such platforms are suitable for experimental work, allowing researchers to establish relationships and effect sizes between different conceptual variables, even if samples are not representative [47]. Although the conclusions drawn are limited, the information is still useful to begin testing hypotheses in diverse samples, making inferences into subpopulation characteristics, and showing that specific phenomena exist [24,48].

A summary of the sampling platforms and methods for online surveys with their respective potential advantages and disadvantages can be found in Table 1.

**Table 1.** Sampling platforms and methods for online surveys with respective potential advantages and disadvantages.

Sampling method and survey platforms		Potential advantages	Potential disadvantages	Implications for research
Probability sampling				
	Random sampling: recruitment through an online sampling frame and randomization of participants	Generalizability of findings to an entire population	An online sampling frame may not be available or obtainable for a given population Not all surveys require populations representation Large probability sampling approaches may lead to underrepresentation of underserved populations	Suitable approach for research among online populations with a defined sampling frame, with a goal of characterizing the true spread or prevalence of phenomena to be investigated
Nonprobability sampling				
	River sampling: recruitment through sites where target populations visit	Cost-effective and convenient approach	Coverage bias: Not every subgroup or target population of interest may be equally represented on digital platforms	Suitable approach for research among harder-to-reach or underserved populations May require additional weighting or offline sampling approaches to better characterize target populations
	Crowdsourcing platforms: recruitment through online marketplaces where potential participants are ‘hired’ to participate in research	Larger sample sizes can be generated quickly compared to other methods	Participant characteristics are not typically curated to be representative of a target population More screening procedures and larger sample sizes are required to sufficiently power analyses	Suitable approach for research that uses inferential statistics, controlling for relevant confounders that are present in a wide range of participants
Either probability or nonprobability sampling depending on recruitment				
	Online panels: recruitment through prerecruited individuals who have agreed to take part in research	Allow for more representative samples through quota sampling Allow for methods that verify the identity of participants	Greater monetary cost In spite of quotas, certain populations remain underserved through online panels Participant pool may reflect only the most active platform users and not all members of online panels	Suitable approach for research among the general population, especially with the potential for government-backed verification and quota sampling Limitations that reflect differences between participants who participate in online panels versus those who don’t need to be accounted for

## Postdata Collection: Strategies to Identify Fraudulent Data

Measures to reduce the inclusion of fraudulent data in online surveys postdata collection involve analyzing metadata to flag suspicious responses and removing low-quality data to ensure the final dataset is more reliable and accurate.

### Metadata Checks: Duplicate Emails

One of the primary methods of metadata checks is to flag responses that share identical email addresses with previously enrolled participants, or similar email addresses with slight variations in the order of the letters or numbers [7,8]. One example removed any email address that had numbers exceeding 4 digits, as it is a sign of a bot-generated email [33]. To further ensure the quality of such checks, researchers can let the email addresses be checked for any discrepancies by a third researcher after conducting the previous 2 steps of removal based on protocol or duplicates [29]. Reimbursements should be done after all the checks have been conducted. In the case that a genuine participant was excluded erroneously, the research team’s contacts should be available for the participant to contact.

### Metadata Checks: IP Addresses and VPN

While IP addresses can be shared among legitimate respondents in communal spaces or households, patterns of identical IP addresses across multiple submissions can indicate fraud. Researchers can consider implementing a feature to flag multiple submissions from the same IP address [33]. However, this alone should not be used as an automatic rejection measure, but considered as an aid in the review of responses for potential data quality issues. Although IP addresses can act as a proxy for the legitimacy of a response, such as the geographic location, it is not completely accurate, as participants can fake their IP address or use the same computer [11].

Furthermore, participants may use a virtual private network (VPN) or a virtual private server to change their IP address [29,31]. The use of VPNs and proxies to mask respondents’ true locations poses a significant challenge in ensuring data validity. Such challenges can be addressed by designing tests to detect discrepancies between the time zone reported by the participant’s browser and the IP address’s inferred time zone [31]. If the time zones differed significantly, the response can then be flagged as potentially suspicious. However, researchers must consider that the average internet user is also more likely to make use of VPNs now, meaning that IP addresses may not necessarily be a signal of a low-quality response [15]. Despite its complications, IP address duplication checks are one of the most common data quality checks [39].

### Metadata Checks: Survey Timing and Duration

Another aspect of metadata checks is the analysis of survey timing and duration. Rapid survey submissions, where multiple surveys are completed within an unrealistically short timeframe, can often indicate bot activity [6,7,11]. Suspicious submissions can be identified by comparing the start and stop times of surveys, flagging those completed within a minute of each other [8]. Survey duration can also be used as a key indicator of fraudulent responses, as overly rapid completion times are unrealistic for genuine participants [39]. Studies have shown that valid respondents typically spend more time on surveys, with response time distributions skewed to the right on a histogram visualization [31]. Pretests or soft launches can help establish realistic time boundaries for survey completion, further enhancing the detection of improbable durations [30,49].

### Data Quality: Duplicate and Improbable Free-Text Responses

By incorporating multiple qualitative questions and making at least one a requirement for survey submission, researchers can more easily identify automatic responses [33]. One possible protocol is that a response is seen as identical if it is repeated 100 times or more for single words, 10 times or more for 2-word entries, and 3 times or more for entries of 3 or more words [8]. In one study, researchers found that qualitative survey questions, despite being optional, were instrumental in identifying 88 (13.3%) bot responses through exact duplicate answers unlikely to occur by chance [29]. The effectiveness of qualitative questions in detecting bots is supported by research, which found that bots struggled with open-ended questions requiring a minimum response length, suggesting that incorporating multiple such questions can enhance bot detection [29].

### Data Quality: Suspicious Response Patterns and Data Inconsistencies

Detecting straight-lining, consistencies, or patterns in the response of a participant across a series of questions is crucial for identifying low-effort or automated responses. Thus, it is important to check for uniform answers across grid questions or obvious patterns, such as sequential responses [8,11]. These patterns can indicate a lack of genuine engagement with the survey content. Similarly, verifying selection patterns in multiple response questions and detecting low differentiation in answers also identifies low-quality responses [38]. Furthermore, detecting inconsistencies in responses helps identify respondents who may not be providing truthful or accurate information [29]. It is important to exclude participants with inconsistent responses, such as mismatched ages and birth dates, as this ensures that survey data accurately represents the target population [11]. For example, one study excluded participants whose answers to questions about sex, gender, and sexuality were contradictory, eg, “I have had insertive vaginal sex with multiple female partners,” yet “none of my partners have vaginas” [11]. Implementing comprehensive checks for straight-lining, response patterns, and internal consistency helps filter out low-quality data, ensuring that the final dataset is reliable and valid.

Fraudulent data can occur through a variety of ways, and thus poses distinct challenges for the prevention and detection of such responses [39]. It is important to consider which methods would be best suited for each study, and using multiple methods in a multipronged approach can help to account for the shortcomings of any single method [8,11]. Researchers can consider taking a dynamic approach with evolving screening protocols based on the type of responses received across the study in order to keep up with ever-changing fraud attempts [38]. In addition, when deciding the threshold required to identify a response as fraudulent or low quality, researchers could assign points to indicators of suspicious activity, which reduces the likelihood of incorrectly excluding valid data [8,33,38]. Having a point system allows specific responses to be flagged for further investigation and establishes a cutoff score for invalid responses [30]. This would also follow best practices in which responses would be removed based on multiple issues rather than a single failure.

## Case Study

An example of a study that was able to maintain a high level of rigor throughout its run was the Restore study by Dewitt et al [38]. The study wanted to recruit gay, bisexual, and other men who have sex with men who were treated for prostate cancer. The challenges they faced with recruitment were because it was a minority within a minority group, and it largely impacted an older demographic, which meant that the process of recruitment had to be as streamlined as possible due to the unclear level of internet familiarity the target population would have. Ultimately, they focused recruitment on a community partner’s email list, who focused on providing support for survivors of cancer, as well as Facebook groups of prostate cancer community organizations, which would be a form of targeted river sampling. They hosted their survey on Qualtrics, a web-based survey platform, and used built-in protection features such as “Prevent Ballot Box Stuffing,” which prevents multiple submissions based on browser cookies, and “Prevent Indexing,” which blocks the survey from being indexed and found on search engines. In addition, they made use of multiple data quality control measures, such as eligibility screeners and manual data validation using both automated and hybrid protocols that flagged suspicious survey entries to the researchers. For example, survey metadata was checked (eg, short response timing, IP addresses that did not match geographic locations), and open-ended responses were evaluated to see if they were reasonable or possible. They ran into issues with a spam attack through the Facebook link and noticed quickly due to a sudden influx of low-quality survey attempts. A further problem was that the answer patterns began to evolve, where initial spam attempts were clearly fraudulent, but later entries began to provide more probable responses. Later entries were only identifiable by manually checking the process analytics and interpreting each survey response’s data. After some review, the researchers created a new copy of the survey and imposed a stricter recruitment protocol with more validation steps and focused their recruitment through community partners’ email lists. They did not provide reimbursement to surveys identified as invalid but provided a method for participants to contact them to verify their answers and receive their compensation if they were unwittingly identified as invalid, but not a single invalid respondent followed up on this. The restore study is a great case study of a dynamic protocol that was able to respond to shifting patterns of fraudulent responses and successfully maximize the rigor of their study.

## Challenges and Opportunities of Artificial Intelligence

One issue of concern is that bots are becoming increasingly sophisticated and are able to better replicate human behavior, allowing them to potentially overwhelm measures put into place and more easily gain access to online surveys [8,50]. CAPTCHAs and open-ended questions can now be solved by high-end bots, and the rise of artificial intelligence (AI) software like ChatGPT means that open-ended questions can also be answered automatically [50,51]. In a study to see if AI-generated texts could be differentiated from actual people, AI-generated texts were only accurately recognized 40.45% of the time, meaning that even manual evaluation of answers may not be sufficient to weed out invalid responses [52]. The level of sophistication that fraudulent responders can achieve using AI and bots only continues to grow, and they pose an incredible threat to data quality when conducting online surveys as they can easily submit large numbers of responses while masquerading as multiple valid respondents [27]. As such, ensuring that surveys do not fall into the wrong hands is even more critical. Researchers should embrace the fact that different sampling methods reach different demographics and even consider multimodal and blended samples where multiple mediums of recruitment and communication complement each other to obtain higher quality samples [24]. Researchers themselves can leverage technological growth, such as by using automated fraud detection systems on web-based survey platforms. For example, Qualtrics includes RelevantID, which uses machine learning to evaluate behavioral data, browser interactions, and other metadata to identify the likelihood of fraud [8]. However, these automated systems should be integrated with manual review or other fraud detection methods to maximize effectiveness.

## Ethical Considerations

In addition, researchers need to consider ethical issues when deciding what data should be collected for screening measures. For example, one of the most common methods used in assessing data quality is to collect IP addresses, or personal details such as email addresses and phone numbers; yet, these undermine one of the key advantages of online surveys, which is anonymity [27]. If a study is particularly sensitive or collects details of criminalized behavior, collecting IP addresses and personal identifiers may introduce additional complications and concerns about data breaches or loss of confidentiality and anonymity. One possible way to overcome this is to have personal details collected in a separate survey, such that key identifiers are not able to be tied to a specific response. Second, the methods reported online are intrinsically limited because studies are unable to definitively categorize which responses are valid or invalid, so we cannot truly evaluate the precision of techniques or their effectiveness. Furthermore, methods are often not verified as per randomized controlled trials and are mostly developed through empirical research when researchers experience fraudulent data in their studies. It would be of value for research to be done comparing different measures against some unfalsifiable assessment of real data in order to accurately determine the effectiveness of such measures. There is still significant room for strategies to develop, and it should be a key focus for upcoming research due to the rise in online surveys being a premier choice for survey-based research.

## Conclusion

Methodological rigor is key for any study regardless of its modality, but online surveys require researchers to consider broader methodological considerations as compared to offline research [53]. Although a myriad of ways in which researchers can increase the rigor of their online surveys are presented in this article, there is no singular “best” method as every research method has its own limitations [54]. Online research appears superficially similar to offline research, but its characteristics affect the entire research process from initial design to reporting the findings in ways that influence their implementation [55,56]. Ultimately, researchers must consider the wide range of factors unique to the requirements of their own studies and make use of a combination of various resources and methods to increase the quality of research published.

## Abbreviations

AI: artificial intelligence
VPN: virtual private network
